# Microbial Proteases in Baked Goods: Modification of Gluten and Effects on Immunogenicity and Product Quality

**DOI:** 10.3390/foods5030059

**Published:** 2016-08-30

**Authors:** Nina G. Heredia-Sandoval, Maribel Y. Valencia-Tapia, Ana M. Calderón de la Barca, Alma R. Islas-Rubio

**Affiliations:** 1 Department of Plant Food Technology, Research Center for Food and Development, A.C. Carretera a La Victoria km 0.6. Hermosillo, Sonora 83304, Mexico; nina_heredia@estudiantes.ciad.mx; 2 Department of Nutrition, Research Center for Food and Development, A.C. Carretera a La Victoria km 0.6. Hermosillo, Sonora 83304, Mexico; m_yael@hotmail.com (M.Y.V.-T.); amc@ciad.mx (A.M.C.d.l.B.)

**Keywords:** gluten-free, baked products, microbial proteases, immunogenicity, product quality

## Abstract

Gluten-related diseases are a range of inflammatory disorders of the small intestine, characterized by an adverse response to gluten ingestion; therefore, the treatment is a gluten withdrawal. In spite of the increased market of gluten-free products, widely available breads with high acceptability are still missing due to the technological challenge of substituting the special gluten properties. Instead of using alternative ingredients for baking, some attempts have been done to decrease gluten immunogenicity by its enzymatic degradation with microbial proteases. Although the gluten immunogenicity reduction has been reached to an acceptable level, some quality parameters of the products are affected. This review focus on the use of microbial peptidases to prepare less immunogenic baked goods and their effect on product quality.

## 1. Introduction

Gluten-related disorders have an estimated global prevalence of around 5% with an ongoing increase in their incidence [1], and they are characterized by intolerance or sensitivity to gluten ingestion. These disorders involve celiac disease and non-celiac gluten sensitivity, with different pathogenic mechanisms resulting in gastrointestinal symptoms such as diarrhoea, gas passing, bloating, vomiting, constipation, and nausea. Extra-intestinal symptoms such as weight loss, anaemia, chronic fatigue, weakness, muscle cramps, migraine, and behavioural changes are also reported [2]. Additionally, there is wheat allergy, which comprises an adaptive immune response to wheat proteins. While some gliadin fractions and other wheat proteins can act as allergens, celiac disease is characterized by intolerance to prolamins in wheat (gliadin), rye (secalin), and barley (hordein). Finally, the wheat molecules involved in non-celiac gluten sensitivity are still unknown.

Gluten withdrawal is the basis for the management of celiac disease and non-celiac gluten sensitivity, as is a wheat-free diet for wheat allergy. Thus, the demand for gluten-free foods has increased in the last decades and baked products are the highest grossing food in the gluten-free market. However, alternatives to gluten, which is the structure-building protein essential for formulating high-quality yeast-raised baked goods, are not easily found [3]. Formerly, the gluten-free baked goods were formulated with starches and hydrocolloids, resulting in poor sensorial properties. In addition, fermented hydrocolloids at the large intestine increase the risk of gastrointestinal symptoms. Nowadays, gluten-free formulations have improved sensory properties.

Different attempts have been made for reduction of immunogenic gluten sequences of wheat while keeping its baking technological properties. In the last decade, several studies have shown the capacity of proteolytic enzymes, mainly peptidases, to degrade gluten during food processing. More recently, it was shown that selected *Lactobacillus* in combination with fungal and/or malt proteases could decrease the residual concentration of gluten immunogenic sequences during extended fermentation times [4,5,6,7]. However, its utilization may affect the technological properties of dough and the quality of baked products. In this review, we focus on the use of peptidases from microbial sources in baked products as an alternative strategy to decrease the immunogenicity of gluten proteins and on their effect on product quality.

## 2. Gluten-Related Disorders and the Gluten-Free Market

The spectrum of gluten-related disorders refers to celiac disease and non-celiac gluten sensitivity, and even to some types of the wheat allergy also exacerbated by gluten proteins, with different mechanisms involved in their pathogenesis. In celiac disease, a T cell–mediated autoimmune reaction is triggered by gluten-derived peptides in genetically predisposed subjects carrying the HLA-DQ2 and/or -DQ8 haplotypes [8]. Wheat allergies are also adverse immunologic although not autoimmune reactions to proteins contained in gluten and other wheat fractions, with an inflammatory response mediated through mast cell activation. A third type of symptomatic response to gluten ingestion is non-celiac gluten sensitivity, which probably involves the immune system, and is diagnosed by exclusion of celiac disease and wheat allergy [2]. Despite the current advances on the pathogenesis of gluten-related disorders, the only approved treatment is still a lifelong gluten-free diet for celiac disease and non-celiac gluten sensitivity, or a wheat-free diet for wheat allergy [2,8].

Gluten-related disorders have gradually emerged as an epidemiologically relevant phenomenon with an estimated global prevalence of around 5% [1]. However, many people perceive gluten as unhealthy, and around 30% of the people in the United States are limiting gluten ingestion [2,9]. This is fuelling a global market of gluten-free products estimated at $8.8 billion by 2014, representing an increase of 63% from 2012 to 2014 [10]. Meanwhile, the latest European reports estimate a compound annual growth rate of 10.4% between 2014 and 2019 [1].

As wheat contains dietary fibre and is fortified with minerals and vitamins, celiac disease patients, especially children, with a gluten-free diet lack such nutrients and are at risk of dietary unbalance. Additionally, they could be psychologically affected due to the drastic dietary changes to gluten-free foodstuffs [11]. Thus, increasing interest in gluten-free products has resulted in the necessity of developing more options and variety to satisfy these needs. In addition to the strict gluten-free diet for celiac patients, some patients suffering gluten sensitivity could benefit from reduced-gluten products. The *Codex Alimentarius Commission* [12] define gluten-free foods as those consisting of ingredients which do not contain wheat, rye, barley, and oats of their crossbred varieties; the gluten level does not exceed <20 mg/kg in total; and foods specially processed to reduce gluten content to a level above 20 up to 100 mg/kg in total, based on the food as sold or distributed to the consumer.

Despite the proven benefits of the gluten-free diet in patients with gluten-related disorders, it may be exceedingly difficult to completely avoid gluten-containing foods, and an effective adherence to the diet has been estimated to be from 56% to 76% [13,14]. Complete exclusion of dietary gluten is difficult due to the ubiquitous nature of this protein complex, cross-contamination of foods, inadequate food labelling regulations, and social constraints [6]. In addition, gluten-free products are a technological and nutritional challenge, considering they are both poor in quality and more expensive than gluten-containing products. Gluten-free products are frequently made using refined gluten-free flours or starches and they are generally not enriched nor fortified like the wheat-based counterparts that they are intended to replace [3]. Therefore, the development of gluten-free products has focused on their technological, sensorial, and nutritional requirements.

Bakery products, which include cookies, crackers, cakes, and other baked goods, are the highest grossing packaged goods in the gluten-free market, with bread as the largest contributor to this market. Gluten-free bread sales showed a seven-fold increase from 2009 to 2011, passing from the eighth to the second place among the gluten-free market of bakery products [9].

## 3. Role of Gluten in Baked Products

Wheat proteins can be classified on the basis of their solubility. The common protein fractions in wheat include albumins (soluble in water and dilute buffers), globulins (soluble in salt solutions), glutelins (soluble in acidic/basic solutions), and gliadins (soluble in 70% alcohol solutions) [9]. Similar proteins have been found in rye and barley. Prolamins (gliadins and glutenins) have a high content of proline (15%) and glutamine (35%) and, depending on the cereal, they have been termed secalin for rye, hordein for barley, avenin for oats, and gliadin for wheat. The high concentration of these amino acids, especially proline, limits proteolysis by gastrointestinal enzymes, preventing the complete degradation by human gastric and pancreatic enzymes. This results in the generation of oligopeptides in the small intestine, which are the main stimulators of the inflammatory response to gluten [6].

In the bakery, gluten is a set of structure-building proteins essential for formulating high-quality yeast-raised baked goods. Traditionally, bread is based on flour derived from common wheat (*Triticum aestivum*). Bread making is a complex process that consists of mixing wheat flour, water, salt, and yeast in varying proportions to form a viscoelastic dough. While the dough is mixed, gluten becomes apparent when the mechanical energy induces conformational changes in hydrated wheat proteins through breakage and formations of both covalent and non-covalent bonds. Air bubbles are incorporated during mixing, and they provide gas nuclei from the carbon dioxide generated by yeast fermentation during proofing. This allows the dough to expand and become a softer, lighter, and palatable product after baking [15].

During baking, dough is transformed into crumb. This involves heat and mass transfer. The two phenomena controlling the formation of crumb are gelatinization of the starch granules in the dough and heat setting or the coagulation of the proteins. As the dough begins to transform into the sponge structure of the crumb, the outer layer of the crumb dries to a crust. The structural elements are a continuous phase of gelatinized starch that is enclosed in a coagulated gluten network. Therefore, gluten is among the elements responsible for the bread’s appearance, texture, and quality [15].

Obtaining high-quality gluten-free bread has become a technological challenge that has led to the search for alternative ingredients, additives, and technologies that can increase the diversity of gluten-free baked products. Thus, the search has focused on complementary strategies aiming to decrease the immunogenic effect of the gluten epitopes. Therefore, the utilization of enzymes, specifically proteases, which have the ability to degrade gluten and other proline peptides into small fragments, diminishing immunogenicity, has been proposed. It has been observed that these proteases may markedly decrease the toxicity of prolamin epitopes [4].

Several studies have shown that proteases decrease the residual concentration of reactive gluten during fermentation [6,16]. In addition, they can replace bisulphite, which was previously used to control consistency through the reduction of glutenin disulphide bonds, while proteolysis breaks down peptide bonds. In both cases, the final effect is a similar weakening of the gluten network [17]. The introduction of these enzymes in the baking process has revolutionized their applications, since they provide an alternative in the manufacture of baked goods for patients with gluten-related disorders [17].

## 4. Microbial Proteases for Gluten Degradation

Gluten degradation by proteolysis has been studied for more than 50 years [18,19]. The principle of the microbial proteases use is that some microbial enzymes, unlike human gastrointestinal proteases, can cleave the peptide bonds next to proline residues, which frequently occur in gluten proteins (10%–15% proline). Thus, gluten proteins could be degraded to small peptides (<nine amino acid residues), with lower immunological activity [20,21]. Therefore, gluten degradation could be performed with the use of a wide range of proteases, especially by prolyl-oligopeptidases or peptidases [4,20,22,23]. These proteases are generally found in plants or microorganisms (e.g., bacterial or fungal).

The review’s focus is on the baking processes involving microbial proteases; therefore, just a short mention of their use as therapeutic tools is provided. From the medical approach, oral therapy with microbial proteases to hydrolyse dietary gluten proteins after ingestion in the gastrointestinal tract has been proposed. The use of oral prolyl peptidases from bacterial sources such as *Flavobacterium meningoseptica, Sphingomonas capsulata, Myxococcus xanthus*, and *Lactobacillus helveticus* has shown some disadvantages due to incomplete gluten degradation, allowing the release of immunogenic peptides. Also, it has been reported that these bacterial enzymes are inactivated by pepsin and low gastric pH [24]. Moreover, the use of fungal proteases has been studied, especially the use of *Aspergillus niger* prolyl-endopeptidase (AnPEP). It has been shown that it effectively degrades the immunogenic gluten peptides in vitro and it is resistant to the acidic conditions in the stomach. However, recent clinical trials confirmed their ineffectiveness as an oral therapy [25,26,27].

From the viewpoint of food technology, the use of microbial proteases in raw material or during food processing has been proposed [21,28]. An advantage of gluten proteolysis before gluten ingestion is the cleavage sites into the proline-rich sequences that could expose new cleavage sites for gastrointestinal and brush-border enzymes, which would further enhance the complete degradation of gluten [21].

Traditionally, there are some sourdough sweet-leavened baked goods obtained by microbial protease fermentation from lactic acid bacteria, such as the Genoese dry biscuit, called lagaccio, and a soft cake from north Italy, panettone. There is also the typical sourdough bread, which is a staple food contributing to cultural identity in sundry diets, mostly in Central and Eastern Europe [29]. All of them involve very long fermentation processes, using lactic acid bacteria (LAB), which provide a sour taste to the product [30]. However, traditional sourdough processes are unsuitable to completely degrade gluten for consumption by celiac patients.

Nowadays, seeking gluten immunogenicity reduction in gluten-reduced baked goods, strategies to degrade wheat gluten by a selected pool of LAB alone or in combination with fungal and/or malt proteases have been recalled. These have been used under specific conditions at long fermentation times and generally in semi-liquid fermentations to produce sourdough baked goods [4,5,6,7,20,31,32]. These treatments have been effective in reducing gluten immunogenicity (in most cases), but some adverse effects in the product have been observed. Table 1 lists studies of gluten modification by microbial and or fungal proteases for baked product development, as well as their scopes in immunoreactive gluten reduction.

### 4.1. Implications on Immunogenicity

Gluten proteins, mainly prolamins, called gliadins in wheat, have a highly antigenic potential. Actually, in silico analyses have shown the presence of more than 60 immunogenic peptides from gluten from the *Triticum* species [33]. One of the most immunogenic peptides studied in celiac disease is the 33-mer peptide, due to its high proline (13 residues) and glutamine (10 residues) content [34], which makes it more resistant to enzymatic proteolysis.

**Figure 1:**
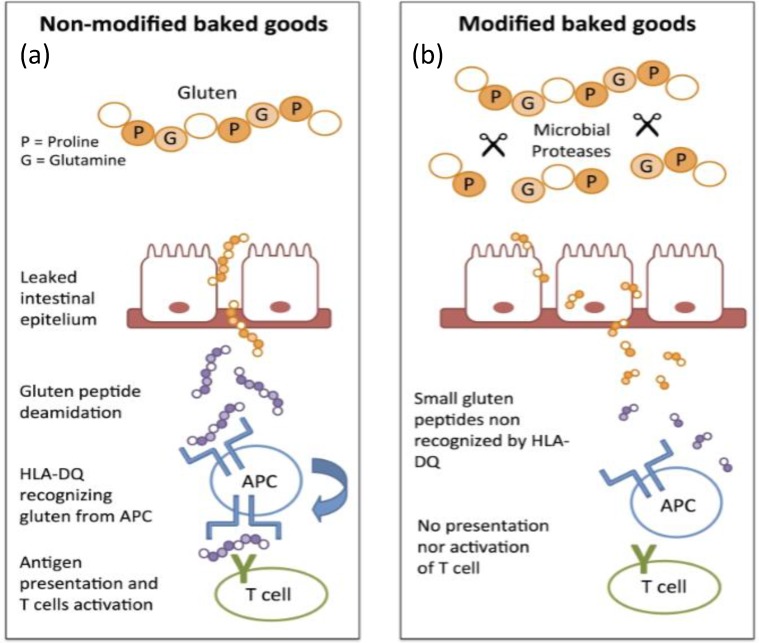
Adaptive immune response to: (**a**) non-modified baked goods; (**b**) and non-activation of T cells to modified baked goods by microbial proteases. Abbreviation: APC: antigen-presenting cell.

There are GRAS (Generally Recognized As Safe) microbial enzymes able to hydrolyse the peptidic bond between proline and other residue such as those in the 33-mer peptide. Figure 1 gives a representation of the possible mechanisms of epitope recognition of this peptide by HLA-DQ2 or DQ8 molecules after deamidation in the lamina propria, in antigen-presenting cells, which is null after hydrolysis [16]. Thus, there is no immunogenic recognition and the pathogenesis of celiac disease is not developed.

The use of LAB during food processing is based on the fact that *Lactobacillus* have a complex protease system, able to hydrolyse various proline-rich peptides, including the 33-mer peptide. Di Cagno et al. [32] described the use of four selected *Lactobacillus* (*L. alimentarius* 15M, *L. brevis* 14G, *L. sanfranciscensis* 7A, and *L. hilgardii* 51B) during wheat flour fermentation, based on the intracellular peptidase activity of these strains, where prolamins almost completely disappeared after 24 h of fermentation. Consequently, the fermented wheat dough (30% *w/w*) was mixed with non-toxic cereals (mix of oat, millet and buckwheat flours) (70% *w/w*). A modified bread was obtained and evaluated in vivo after a challenge of two days of bread consumption (2 g of gluten/day), affirming the treatment’s effectiveness based in the observed response on intestinal permeability of celiac disease patients. However, it is very difficult to predict the toxicity of the product with an in vivo test of such short duration, being an indirect measure for intestinal permeability.

The elaboration of sourdough breads using LAB represents a good way to reduce gluten immunogenicity from gluten-containing flours. However, there is no a unique bacterial strain with the endo- and exo-proteases required to hydrolyse the total gluten polypeptides, because they are so diverse and contain proline residues at different positions [5].

A complete gluten degradation could be possible after the addition of fungal proteases, besides LAB. Fungal proteases such as *A. niger* or *A. oryzae*, routinely used in conventional bread making and considered as food-grade enzymes, have been studied [4,5,6,7,22,23]. An example is the addition of fungal proteases to start the primary proteolysis of gluten, followed by a secondary proteolysis by LAB. At the fermentation system, polypeptides from four to 40 amino acids (including the 33-mer), generated after primary proteolysis, are transported into the bacillus cytoplasm and subjected to a secondary hydrolysis [4,6].

Rizzello et al. [4] showed the effective use of sourdough *Lactobacillus* (*L. sanfranciscensis* LS3, LS10, LS19, LS23, LS38, and LS47) in combination with fungal proteases (*Aspergillus oryzae* and *A. niger*) to eliminate the immunogenicity of wheat flour after 48 h fermentation. A sourdough with 12 mg/kg of reactive gluten was produced and its peptic-tryptic digest was evaluated in vitro by the proliferation of peripheral blood mononuclear cells and their gamma interferon production as well as by intestinal T-cell lines from 12 celiac patients. The proteins extracted from sourdough induced the same release of gamma interferon and activation of mononuclear cells as was done by the culture medium alone. Additionally, the intestinal T-cell lines did not show immunoreactivity to the digestion products.

Greco et al. [22] evaluated the safety of the daily administration of baked goods made from sourdough wheat flour, previously described by Rizzello et al. [4]. The evaluation was done by clinical and serological changes in celiac patients (*n* = 5), after consumption of 200 g baked goods/day for 60 days. No clinical complaints or serological changes were detected in celiac patients. The quality of the obtained baked goods was poorly described, with just a mention about the specific volume being lower than that of a baker’s yeast bread and a typical flavor of sourdough wheat bread.

Recently, Walter et al. [20] used AnPEP to degrade gluten in rye flour and sourdough, to produce sourdough bread. Rye proteins have a minor role in the dough rye structure, where arabinoxylans (up to 7%) plays the main role in the baking performance, providing a higher water absorption, dough viscosity and gas retention capacity. The double addition of AnPEP after 0 and 24 h of fermentation was necessary to fully hydrolyse gluten in rye flour (below 20 mg/kg). However, the presence of arabinoxylans was not sufficient to give a strong dough structure, and gluten degradation negatively affected the rye bread quality.

The effectiveness of LAB and fungal peptidases in the degradation of wheat gluten has been demonstrated, but the acidic flavor and other characteristics, such as the volume of sourdough baked goods, are not well accepted in different cultures [35]. However, no attempts have been done to apply the sourdough fermentation in combination with fungal proteases to the production of sweet-leavened baked goods such as panettone and lagaccio [30,36].

In addition to bread making and other baked goods, wheat is a the source of important ingredients in the food industry, such as starch, bran, and others; such products need to be treated for removing reactive gluten and to prepare gluten-free foods. Walter et al. [37] proved the AnPEP’s effectiveness in achieving the degradation of gluten in wheat starch, wheat bran and bread drink to a concentration of <20 mg/kg.

### 4.2. Implications on Product Quality

In the bread-making industry, the addition of enzymes to the flour or dough is a common practice to improve the dough rheology. Proteases are routinely used on a large commercial scale in the production of baked goods, crackers, and waffles, and their addition levels are usually very low. These enzymes have a great impact on dough rheology and bread quality because of the effects on the gluten network [17]. The use of microbial proteases has been proposed to fully degrade the immunogenic gluten in raw material or during baking processes, which has an effect on dough rheology, due to the gluten’s role in determining the viscoelastic and processing characteristics of the dough [38].

The most studied process for gluten degradation during bread making is sourdough fermentation [4,7,20]. Sourdough is a mixture of flour and water that is fermented with LAB and yeasts (commonly *Saccharomyces cerevisiae*). The proteolytic activity of LAB enzymes to degrade gluten during dough mixing and fermentation may be attributed to the proteolytic activity of LAB and endogenous proteases of flour under acidic conditions. This results in a weaker dough and a decrease in the loaf specific volume; these effects are accentuated when long fermentation times are used [38]. In contrast to traditional sourdough processes, it has been reported that for total gluten degradation, long fermentation times are needed (approximately 24–72 h). The use of sourdough fermentation for bread making plays a crucial role in the development of sensory properties such as taste, aroma, texture, and overall quality of baked goods. This is due to the acidification, proteolysis, and activation of a number of enzymes [17,39].

The combined use of fungal proteases and sourdough fermentation improves gluten degradation, especially in the gliadins fraction [6,7], which are fully hydrolyzed to smaller peptides (<nine amino acids residues). Gliadins degradation is an important factor in dough functionality, due to the proteins' capacity to impart plasticity, extensibility, and viscous properties to wheat flour dough, whereas glutenins are mostly responsible for the elasticity and cohesive strength of the dough [17]. Rizzello et al., [7] showed that when LAB and AnPEP degradation of gluten was efficient, it resulted in a greater decrease of the specific volume and overall acceptability of the modified breads evaluated.

Walter et al. [20] tested the use of AnPEP in rye flour and rye sourdough with an effective degradation of the immunogenic gluten (<20 mg/kg) in both systems. For bread making, they added egg whites as a structuring agent for dough formation. In terms of bread quality, a higher specific volume was obtained with the AnPEP-modified flour in comparison with the combination of AnPEP and sourdough modification (2.2 vs. 1.6 mL/g). Also, the treated sourdough with AnPEP had inferior dough properties, with a low viscosity and high stickiness during proofing, compared to the untreated sourdough. This behaviour could be due to the pH reduction by the sourdough fermentation, inducing an increase in electrostatic repulsion forces. This improves the proteins’ solubility and prevents the formation of new bonds, affecting the final viscosity of the system [40]. The baking performance of the AnPEP bread was better than that of the sourdough bread, but the addition of egg white to improve the dough properties was necessary.

Generally, in gluten-free bread making, some ingredients are incorporated to improve the rheological behaviour of modified doughs. Those ingredients involve the use of starches, hydrocolloids (especially gums), animal and vegetable protein supplements, and gluten-free flours such as rice, soy, amaranth, buckwheat, chickpea, or corn. However, more research is needed to mimic the unique properties of gluten in bakery products.

## 5. Conclusions

Due to the growing trend of the gluten-free market, the development and modification of technologies for the production of reduced or gluten-free products have been studied in the last decade. Nonetheless, the current gluten-free products available on the market have technological and sensory deficiencies due to the absence of gluten. To reduce or eliminate immunogenic gluten, especially in baked goods, the use of microbial proteases during bread-making processes has emerged. Despite the advantages of hydrolysing immunoreactive gluten, these modifications alter the quality characteristics of the final products, especially the specific volume of bread. Therefore, more research is needed to develop baked goods with less immunogenic gluten and good quality.

## Figures and Tables

**Table 1:** Summary of studies on gluten modification by microbial proteases for baked product development.

Target of Modification	Microbial Enzyme Source	Gluten Content after Modification	Reference
Wheat flour	*Lactobacillus alimentarius*, 15M, *L. brevis* 14G, *L. sanfranciscensis* 7A, *L. hilgardii*	ND ^1^	[32]
Wheat flour	*Lactobacillus alimentarius*, 15M, *L. brevis* 14G, *L. hilgardii*, *L sanfranciscensis* (7A, LS3, LS10, LS19, LS23, LS38, LS47); *Aspergillus oryzae* and *A. niger*	<12 mg/kg (in sourdough).	[4]
Wheat flour	*Enterococcus faecalis* G32, ND3 and HM3C; *Rhizopus oryzae* CH4	1106 mg/kg (in treated flour)	[28]
Wheat flour	*Lactobacillus sanfranciscensis* (7A, LS3, LS10, LS19, LS23, LS38, and LS47), *L. alimentarius*, *L. brevis* 14G, *L. hilgardii* 51B; *Aspergillus oryzae* and *A. niger*.	<10 mg/kg (in sweet baked goods).	[22]
Wheat flour	Pool 1: *Lactobacillus alimentarius* 15M, *L. brevis* 14G, *L sanfranciscensis* 7A, *L. hilgardii*; Pool 2: *L. sanfranciscensis* (LS3, LS10, LS19, LS23, LS38, LS47); Fungal proteases: *Aspergillus oryzae* and *A. niger*.	2480 mg/kg (Pool 1) and <10 mg/kg; (Fungal proteases, Pool 1 and 2) (in biscuits and cakes).	[23]
Wheat flour	*Lactobacillus sanfranciscensis* (7A, LS3, LS10, LS19, LS23, LS38 and LS47), *L. alimentarius* 15 M, *L. brevis* 14G, and *L. hilgardii* 51B; *Aspergillus oryzae* and *A. niger*.	20000–76431 mg/kg (in sourdough bread).	[7]
Rye flour	*Lactobacillus brevis and A. niger*.	8–532 mg/kg (in rye treated flour).	[20]

^1^ ND: Not determinated.

## Abbreviations

The following abbreviations are used in this manuscript:

AnPEP	*Aspergillus niger* Prolyl-Endopeptidase
LAB	Lactic Acid Bacteria
APC	Antigen Presenting Cell
GRAS	Generally Recognized As Safe
